# Acetaminophen Poisoning: Case Reports of Early Liver Dialysis for Hepatic Injury

**DOI:** 10.1002/ccr3.70642

**Published:** 2025-07-17

**Authors:** Mitra Rahimi, Shahin Shadnia, Babak Mostafazadeh, Peyman Erfan Talab Evini, Sayed Masoud Hosseini, Niloofar Deravi, Mohaddeseh Belbasi

**Affiliations:** ^1^ Toxicological Research Center, Excellence Center of Clinical Toxicology, Department of Clinical Toxicology, Loghman Hakim Hospital Shahid Beheshti University of Medical Sciences Tehran Iran; ^2^ Students Research Committee Shahid Beheshti University of Medical Sciences Tehran Iran; ^3^ Students Research Committee, School of Pharmacy Zanjan University of Medical Sciences Zanjan Iran

**Keywords:** acetaminophen, artificial liver support, case reports, liver function tests, poisoning

## Abstract

In acetaminophen‐induced acute liver failure, early use of extracorporeal artificial liver support using BS330/HA330‐II filters improves liver function, coagulation profiles, bilirubin levels, and may obviate the need for liver transplantation. Early adjunctive use with N‐acetylcysteine may optimize outcomes; further studies are necessary to confirm efficacy, safety, and standardized protocols.

## 1 Introduction

Acetaminophen, also known as paracetamol or APAP, is a nonopioid analgesic and antipyretic. The medication is available in immediate or extended‐release formulations, either as a standalone product or in combination with other medications such as codeine or ibuprofen [1]. The widespread availability of over‐the‐counter and prescription products containing acetaminophen has contributed to global concern regarding its toxicity [2]. The management of acute deliberate self‐poisoning, accidental pediatric exposure, and inadvertent repeated supratherapeutic ingestions necessitates the implementation of distinct risk assessment and management strategies. Important considerations for paracetamol poisoning include the type and amount of paracetamol ingested, the time elapsed since ingestion, and the level of paracetamol in the bloodstream. Early assessments should focus on serum paracetamol concentrations, while late assessments should consider clinical and laboratory signs of acute liver injury. The serum paracetamol concentration is crucial for determining the necessity of administering N‐acetylcysteine (NAC) [3]. NAC is widely regarded as the primary antidote for acetaminophen poisoning [4]. The administration of NAC is recommended when the acetaminophen level indicates potential toxicity according to the Rumack‐Matthew acetaminophen treatment nomogram. The protective effects of NAC are most pronounced when it is administered within 8 h of ingestion [5]. However, its efficacy appears to be limited in severely ill patients who present 24 h after acetaminophen ingestion [6].

Patients who present 24 h after an acetaminophen overdose are at a heightened risk of hepatic failure, with a 25% mortality rate despite acetylcysteine therapy. When patients meet the King's College criteria for liver transplantation, the mortality rate increases from 60% to 80%. Even with a liver transplant, the one‐year mortality rate remains at 20%–30%. Given the high risk of hepatic necrosis in acetaminophen overdose patients, extracorporeal detoxification appears to be a promising treatment approach [7].

The Molecular Adsorbent Recirculating System (MARS) and the double plasma molecular adsorption system (DPMAS) are types of medical devices that are classified as part of the artificial liver support system [8]. DPMAS is a new method for artificial liver support that includes two separate filters to remove toxins from the patient's circulatory system. In this technique, the plasma filters are connected in series, which results in more effective cleaning of toxins during the dialysis process. First, the patient's blood enters the first blood pump, and then the blood plasma is separated by the plasma separator filter and enters the plasma pump. At this stage, the plasma enters the double cartridges BS330 and HA330‐II. Finally, pure plasma returns to the patient's blood circulation [9, 10]. MARS has a different treatment approach. This system uses albumin as a detoxification agent. Dialysis fluid enriched with albumin is used in this technique. Then, this liquid enters the device to exchange with plasma toxins. Finally, the purified blood returns to the patient's circulation [11].

BS330 and HA330‐II cartridges each target specific toxins. BS330 specifically removes bilirubin, bile acid, and endotoxins. On the other hand, HA330‐II works against a wider range of toxins, such as inflammatory factors, ammonia, and phenol mercaptan [12]. DPMAS has been found to be well tolerated, and prior research has shown its efficacy in the management of acute liver failure [13, 14]. Reporting the results of using this method in patients can help determine its effectiveness and safety. Here, we describe three instances of acetaminophen toxicity that were managed using extracorporeal artificial liver support. It was observed that extracorporeal artificial liver support successfully improved liver function tests in all patients.

## 2 Case History/Examination

### 2.1 Patient 1

An Iranian 16‐year‐old male adolescent presented to our emergency department after an intentional overdose of one hundred 500 mg tablets of acetaminophen more than 15 h prior (November 27, 2023). On presentation, the patient was obeying and oriented with a GCS score of 15, and his vital signs were normal. His vital signs at presentation were as follows: heart rate of 78 beats/min, respiratory rate of 16 breaths/min, body temperature of 37.1, and blood pressure of 119/81 mmHg. The pupils were mid‐sized (3–4 mm), symmetric, and light reactive. The patient had no history of suicide, psychiatric illness, or substance abuse. The patient had a history of G6‐PD deficiency and impaired LFTs.

### 2.2 Patient 2

The patient, a 27‐year‐old Iranian single female, was referred to the emergency room of this center 5 h after consuming 70 tablets of 500 mg of acetaminophen (December 30, 2023). In the initial examination, the pupils were medium‐sized and reactive to light. The GCS score of the patient was 14. Primary vital signs included a blood pressure of 130/70, a respiratory rate of 16, a heart rate of 75, a temperature of 36.8, and SpO2 of 99%. Other examinations were normal. The patient had no history of substance abuse, suicide, or mental illness.

### 2.3 Patients 3

A 51‐year‐old Iranian married female patient was referred to the emergency department of this center after taking an unknown number of acetaminophen and digoxin tablets for more than 12 h (December 31, 2023). At the time of the visit, she had stable vital signs, as follows: blood pressure of 124/90, respiratory rate of 12, and heart rate of 80. The pupils were pinpointed and did not react to light. Her initial GCS score was 14. The patient had a previous history of substance abuse, suicide, and psychiatric illness.

## 3 Differential Diagnosis, Investigations, and Treatment

### 3.1 Patient 1

Immediately after hospitalization, the patient's serum acetaminophen level was checked and reported as 89 μg/mL. Therefore, 7 g of NAC TDS was administered. Due to increasing LFTs and bilirubin levels despite routine treatment, artificial liver support was provided to the patient (November 29, 2023). First, a femoral Shaldon catheter was implanted in the patient. Then, the patient was connected to the first blood pump for 2 h, and 15 mL/kg, equivalent to 750 cc of plasma, was exchanged. Four units of FFP and two albumin vials were prescribed to the patient. Conventionally, these drugs are administered during dialysis. However, at the end of therapy, the patient presented with stable hemodynamic and vital signs, and no norepinephrine intake. This procedure was used to purify the contaminated plasma as much as possible. In the second stage of hemodialysis, the patient was connected to the second blood pump, which included the hemoperfusion device, through the second line. The patient underwent plasma filtration for 5 h through the BS330 cartridge. Moreover, the patient's blood and plasma entered the patient's blood circulation through the venous chamber of the first pump. Finally, approximately 100 cc of plasma bound to the filter. Notably, we did not utilize the HA330‐II cartridge in this patient.

### 3.2 Patient 2

Before visiting this center, the patient had been treated with charcoal and an NG tube. The level of acetaminophen in the patient's blood was reported to be 240 μg/mL, and she was treated with NAC. On liver ultrasound, parenchymal echo was slightly increased in favor of Grade 1 fatty liver. Due to the increasing liver enzymes in spite of routine treatment and high serum level of acetaminophen, the patient underwent artificial liver support according to the method mentioned in the above section (January 03, 2024). Due to the patient's low bilirubin level, artificial liver support was conducted using only the HA330 cartridge.

### 3.3 Patient 3

In the UDT test, opium and BZD were positive. The serum acetaminophen level was reported as 108 μg/mL after 24 h of taking the drug, and the patient was treated with 6 g of NAC three times a day. On the third day of hospitalization, the patient was intubated.

Due to the increasing liver enzymes in spite of NAC therapy and high serum level of acetaminophen, even after 24 h post‐ingestion, the patient underwent artificial liver support for 4–6 h according to the abovementioned method (January 04, 2024). In this patient, due to the patient's low bilirubin level, artificial liver support was provided using only the HA330 cartridge.

## 4 Outcome and Follow‐Up

### 4.1 Patient 1

**FIGURE 1:**
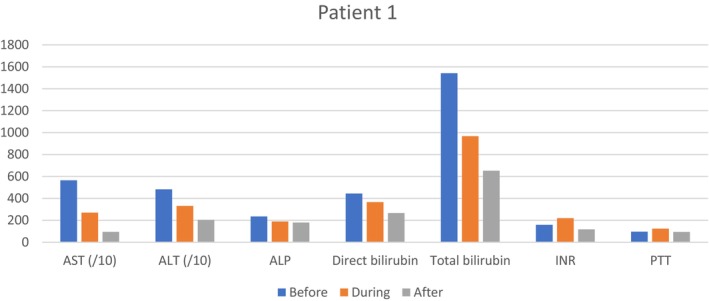
Laboratory test results for patient No. 1 depicted as bar graphs with Y axis %normal. The upper limit of variables normal range used for calculation was as follows: AST: 40 IU/L; ALT: 56 IU/L; ALP: 120 IU/L; INR: 1.2; PTT: 35 s; Direct Bilirubin: 0.3 μmol/L; Total Bilirubin: 1.2 μmol/L. For a better visualization of the graph and to bring the values of different variables closer together, the AST and ALT values were divided by 10.

Abdominal and pelvic ultrasound revealed a liver with normal dimensions and a brief echo of heterogeneity; other parameters were reported to be normal. Additionally, the liver function test parameters improved (Table 1 and Figure 1). After developing oral tolerance, the patient was discharged with good general condition and stable vital signs (December 03, 2023). Warning signs, including fever, dizziness, severe vomiting, severe pain, and bleeding, as well as psychiatric instructions, were provided.

**TABLE 1:** Laboratory test results for patient No. 1.

Variable	Before artificial liver support	During artificial liver support	After artificial liver support
Aspartate aminotransferase (UI/L)	208, 135, 6401, 2798, 1756	1080	996, 592, 328, 184, 112, 61
Alanine aminotransaminase (IU/L)	307, 112, 5211, 4306, 3584	1858	1831, 1503, 989, 1144, 860, 547
Alkaline phosphatase (UI/L)	264, 303, 280	227	206, 188, 224, 245
International normalized ratio	1.07, 2.41, 2.51, 1.67	2.64	2.43, 1.5, 1.3, 1.14, 1.04, 1.08
Partial thromboplastin time (s)	31.2, 37.8, 34.6, 36.1, 30	43.6	37, 31, 34.1, 33.3, 35.7, 25, 34
Direct bilirubin (μmol/L)	1.1, 1.3, 1.6	1.1	1, 0.6, 0.8, 0.9, 0.7
Total bilirubin (μmol/L)	19.9, 18.6, 18.2, 17.3	11.6	12.6, 9.4, 8.2, 7.6, 5.5, 3.7

### 4.2 Patient 2

**FIGURE 2:**
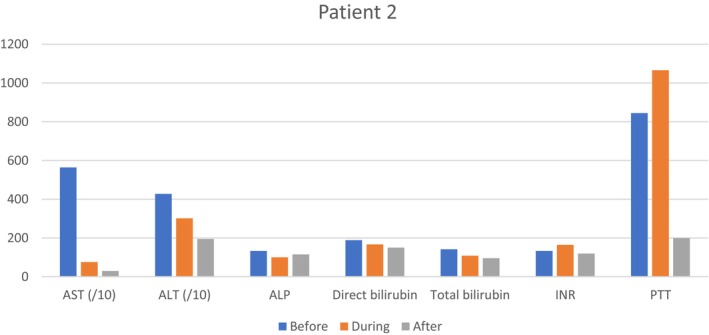
Laboratory test results for patient No. 2 depicted as bar graphs with Y axis %normal. The upper limit of variables normal range used for calculation was as follows: AST: 40 IU/L; ALT: 56 IU/L; ALP: 120 IU/L; INR: 1.2; PTT: 35 s; Direct Bilirubin: 0.3 μmol/L; Total Bilirubin: 1.2 μmol/L. For a better visualization of the graph and to bring the values of different variables closer together, the AST and ALT values were divided by 10.

The patient's symptoms and laboratory parameters improved during hospitalization (Table 2 and Figure 2). Finally, despite the recommendation to continue the treatment, the patient was discharged with personal consent (January 06, 2024). Warning signs and red flags were explained to her.

**TABLE 2:** Laboratory test results for Patient No. 2.

Variable	Before artificial liver support	During artificial liver support	After artificial liver support
Aspartate aminotransferase (UI/L)	389, 2488, 2589, 3559	302	247, 61, 44
Alanine aminotransaminase (IU/L)	271, 175, 3036, 5930, 2553	1687	1346, 995, 937
Alkaline phosphatase (UI/L)	174, 171, 158, 135	120	138, 138
International normalized ratio	1.3, 1.8, 1.7	1.97	1.55, 1.46, 1.3
Partial thromboplastin time (s)	60, 23, 804	373	146, 36, 47, 50
Direct bilirubin (μmol/L)	0.5, 0.6, 0.6	0.5	0.5, 0.4
Total bilirubin (μmol/L)	1.7, 1.8, 1.6	1.3	1.3, 1

### 4.3 Patient 3

**FIGURE 3:**
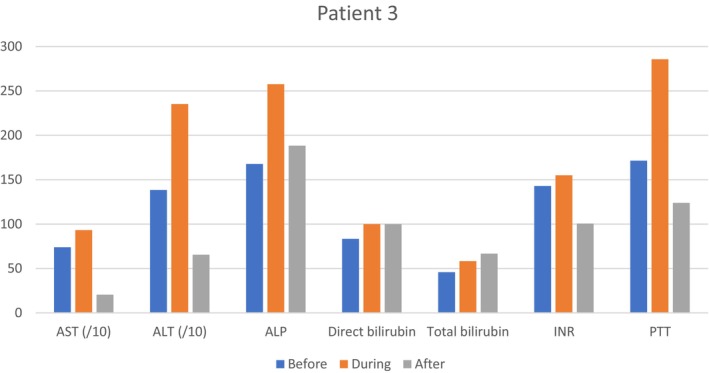
Laboratory test results for patient No. 3 depicted as bar graphs with Y axis %normal. The upper limit of variables normal range used for calculation was as follows: AST: 40 IU/L; ALT: 56 IU/L; ALP: 120 IU/L; INR: 1.2; PTT: 35 s; Direct Bilirubin: 0.3 μmol/L; Total Bilirubin: 1.2 μmol/L. For a better visualization of the graph and to bring the values of different variables closer together, the AST and ALT values were divided by 10.

On January 12, 2024, after liver function tests, coagulation profile improvement, and oral tolerance establishment, the patient was discharged with good general condition and stable vital signs (Table 3 and Figure 3). The red flags were explained to her.

**TABLE 3:** Laboratory test results for Patient No. 3.

Variable	Before artificial liver support	During artificial liver support	After artificial liver support
Aspartate aminotransferase (UI/L)	60, 23, 804	373	146, 85, 50, 47
Alanine aminotransaminase (IU/L)	68, 17, 2241	1317	852, 280, 194, 141
Alkaline phosphatase (UI/L)	167, 148, 180, 230, 281	309	250, 232, 213, 209
International normalized ratio	1.25, 1.97, 1.76, 1.88	1.86	1.24, 1.14, 1.24
Partial thromboplastin time (s)	39, 41, 100	100	53.7, 33
Direct bilirubin (μmol/L)	0.3, 0.2	0.3	0.3, 0.3
Total bilirubin (μmol/L)	0.7, 0.4	0.7	0.6, 1

## 5 Discussion

This case report describes three patients with acetaminophen poisoning who were treated with the DPMAS for extracorporeal liver support. Depending on their liver function test results, patients received either a BS330 filter (for elevated bilirubin) or an HA330‐II filter (for impaired LFTs with normal bilirubin levels). Despite severe acetaminophen toxicity and LFT abnormalities that were refractory to conventional therapy, all three patients recovered after artificial liver support.

Excessive acetaminophen can lead to severe hepatotoxicity. An acetaminophen dose more than 7.5–10 g or > 150 mg/kg in adults and > 200 mg/kg in children is considered a toxic dose [15]. The metabolism of acetaminophen occurs through glucuronidation and sulfuration in the liver. In the case of an acetaminophen overdose, these pathways are saturated, and shunted metabolism by CYP2E1 leads to the production of toxic metabolites like NAPQI, which in turn cause irreversible liver necrosis [16]. Acetaminophen poisoning is diagnosed through its serum level. In addition, liver and coagulation profile tests help diagnose acetaminophen toxicity and liver failure [17].

The treatment plan for acetaminophen poisoning is closely related to the time of presentation after drug ingestion. In the first 1 h after ingestion, noncomplex treatments such as decontamination of the digestive system and the swallowing of charcoal can be used [18]. Another efficient treatment is NAC, which has indications, and its injectable form is preferable to oral NAC. These indications include (1) being in the toxic range of the Rumack‐Matthew nomogram, (2) having a serum acetaminophen level > 10 μg/mL and an unknown ingestion time, (3) having an acetaminophen dose > 140 and an ingestion time > 8 h, (4) having abnormal test results and an ingestion time > 24 h, and (5) having any liver damage [19]. NAC administration should be continued for at least 72 h until liver failure, recovery, liver transplantation, or death [20]. Creatine levels > 3.4, PT > 1.8, INR > 6.5, encephalopathy grade 3 or 4, and arterial pH < 7.3 are associated with poor prognosis [21].

Fulminant liver failure accompanied by progressive encephalopathy and coagulation disorders makes patients candidates for liver transplantation. However, until liver transplantation, extracorporeal artificial liver support can be suitable for patients [22]. The indications for this technique include hepatic encephalopathy of at least West Haven grade 2, increased bilirubin, and an INR > 1.5. This treatment is often used as a support system for patients on the liver transplant list, and despite the reduction in bilirubin, it does not affect the mortality rate of all causes [23].

Patient No. 1, with elevated LFTs and bilirubin level, refractory to the routine treatment, was placed on the priority list for artificial liver support to eliminate the need for liver transplantation. DPMAS could have been a bridge to the naturally occurring recovery process of the liver in our patient. Patient 1 only received BS330 because of the elevated bilirubin level, while patients 2 and 3 with normal bilirubin levels only received HA330‐II. The HA 330‐II cartridge, with a neutral macroporous resin, effectively adsorbs middle‐molecular‐weight toxins associated with liver disorders, including inflammatory mediators. While the BS330 cartridge, equipped with an anion‐exchange resin, selectively targets bilirubin and bile acids [24]. Due to these distinct adsorption properties, Patient 1 (with elevated bilirubin) was treated with BS330, while Patients 2 and 3 (with normal bilirubin levels) received HA330‐II. Financial constraints prevented the simultaneous use of both cartridges.

The use of dual cartridges BS330 and HA330‐II, which are specific for water‐soluble toxins and protein‐bound toxins, respectively, followed a targeted approach. This system helps reduce the progression of liver failure and improve outcomes by increasing the accuracy of detoxification. In a study conducted by Sazonov et al. [25], an HA330 cartridge was used for blood purification in three children suffering from sepsis. In these patients, renal function and inflammatory parameters improved significantly after 1–2 sessions of hemoperfusion for 4 h. Finally, all three patients were stabilized. In another case report by Ng et al. [26], extracorporeal artificial liver support was performed for a patient with fulminant liver failure after poisoning with trimethoprim‐sulfamethoxazole. The liver parameters of this patient improved significantly, and he no longer needed a liver transplant. In another case report by Ramezani et al. [27], a case of liver failure due to multidrug poisoning with colchicine and acetaminophen was reported. Eight‐hour hemodialysis‐hemoperfusion was performed for the patient using an HA330 cartridge. The patient's liver parameters improved significantly, and finally, the patient was discharged in good general condition without the need for a liver transplant.

However, this technology also has limitations. According to the manufacturer's protocol, these filters have a 12‐h efficiency. However, we observed that the filter performance significantly decreased after 3–4 h of dialysis due to physical saturation. Additionally, the outcomes of patients who underwent therapy for 8–10 h were not much different from those of patients who underwent therapy for 3–4 h. Therefore, prolonged treatment is unnecessary, but if extended therapy is required, the filter must be replaced with a new one. On the other hand, the long duration of dialysis itself brings risks. Patients need to receive heparin for dialysis, and continued treatment leads to an increased risk of bleeding.

## 6 Conclusion

In conclusion, extracorporeal artificial liver support can improve liver damage caused by poisoning with various drugs. We believe that early dialysis is crucial for patient survival. Using dedicated cartridges (e.g., BS330 and HA330‐II) may enhance liver function, improve coagulation parameters, and lower bilirubin levels, potentially eliminating the necessity for liver transplantation. The performance, efficiency, and safety of this type of treatment should be considered in future studies.

## Author Contributions

**Mitra Rahimi:** conceptualization, writing – original draft, writing – review and editing. **Shahin Shadnia:** writing – original draft. **Babak Mostafazadeh:** conceptualization, writing – original draft, writing – review and editing. **Peyman Erfan Talab Evini:** writing – original draft. **Sayed Masoud Hosseini:** writing – review and editing. **Niloofar Deravi:** writing – original draft. **Mohaddeseh Belbasi:** writing – original draft.

## Ethics Statement

The article is a case report; therefore, approval from the Ethics Review Board at Shahid Beheshti University of Medical Sciences (SBMU) was waived.

## Consent

Written informed consent has been taken from all the patients mentioned in the case series.

## Conflicts of Interest

The authors declare no conflicts of interest.

## Data Availability

The data that support the findings of this study are available from the corresponding author upon reasonable request.
